# Supplementary material for: Single-Molecule Real-Time Sequencing for Identifying Sexual-Dimorphism-Related Transcriptomes and Genes in the Chinese Soft-Shelled Turtle (Pelodiscus sinensis)
Source: Animals (Basel). 2023 Nov 29;13(23):3704. doi: 10.3390/ani13233704 (PMC10705357; doi:10.3390/ani13233704)
Supplement: Supplementary file 1 [file animals-13-03704-s001.zip › Supplementary Tables.pdf]

Table S1 CCS data volume statistics

Library	Total Bases(bp)	ROI	Mean Length (bp)	Mean quality	Mean Passes
F 0-2K	855,098,963	427,494	2,000	0.97	14
F 2K+	1,154,409,533	337,154	3,423	0.95	7
M 0-2K	754,494,230	400,518	1,883	0.96	13
M 2K+	1,160,635,111	371,683	3,122	0.95	8
Total	3,924,637,837	1,536,849	-	-	-

Table S2 The annotation of gene structure

Type	Number of annotated	Number of Identification
Loci	23,371	30,670
Loci<1k	5,560(23.79%)	2,096(6.83%)
Loci 1-2k	5,909(25.28%)	8,975(29.26%)
Loci 2-3k	4,297(18.39%)	8,628(28.13%)
Loci >3k	7,605(32.54%)	10,971(35.77%)

Table S3 Primers for RT-qPCR

Gene	Primer(5'~3')
Smad4	F:ACCAGCACTGCCAACTTTCCC<br>R:GCTGAGGCCCTGATGCTATCTG
Wif1	F:AACCGTCAATGTCCCTCTGCT<br>R:CATCCACTTCAAATGCTGCCAC
Nkd2	F:AGGGTGGCTGACAGGAGGTTG<br>R:GCGAGGTCCAGGTAGTGGTTTCT
17β-hsd	F:ATGCCACATTGCGGGACCTGA<br>R:CACAGCACCCACAGCGTTCAA
CPY2J	F:CCACAACTCTGCGATGGGCTCT<br>R:CGCCGTCAAATTGGTCAGGATC
Neurl1	F:TGAGGAGTTTGCCAATGAGGG<br>R:AGTTGAGTGAGTTCTGCGGGAT
Prp18	F:CGCTCCAAACAACAGAGTCGC<br>R:GTCCTCCTCTTCCTCATACTCCACATC
Wdr24	F:GCAGGTGATGCTGTGGAGTCTT<br>R:AATGGGCTAGGGCAGGGTTTA
