# Supplementary figures and images for: Single-Molecule Real-Time Sequencing for Identifying Sexual-Dimorphism-Related Transcriptomes and Genes in the Chinese Soft-Shelled Turtle (Pelodiscus sinensis)
Source: Animals (Basel). 2023 Nov 29;13(23):3704. doi: 10.3390/ani13233704 (PMC10705357; doi:10.3390/ani13233704)

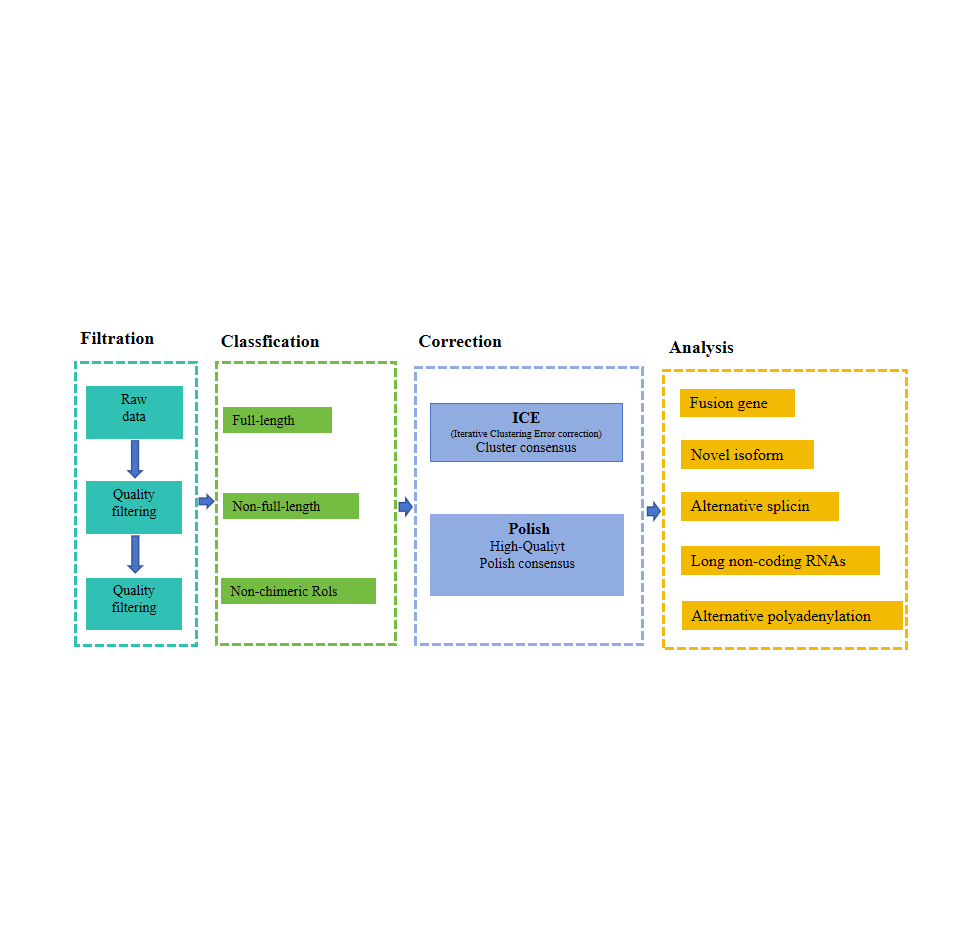

Supplement: Supplementary file 1 [file animals-13-03704-s001.zip › Figure S1.png]

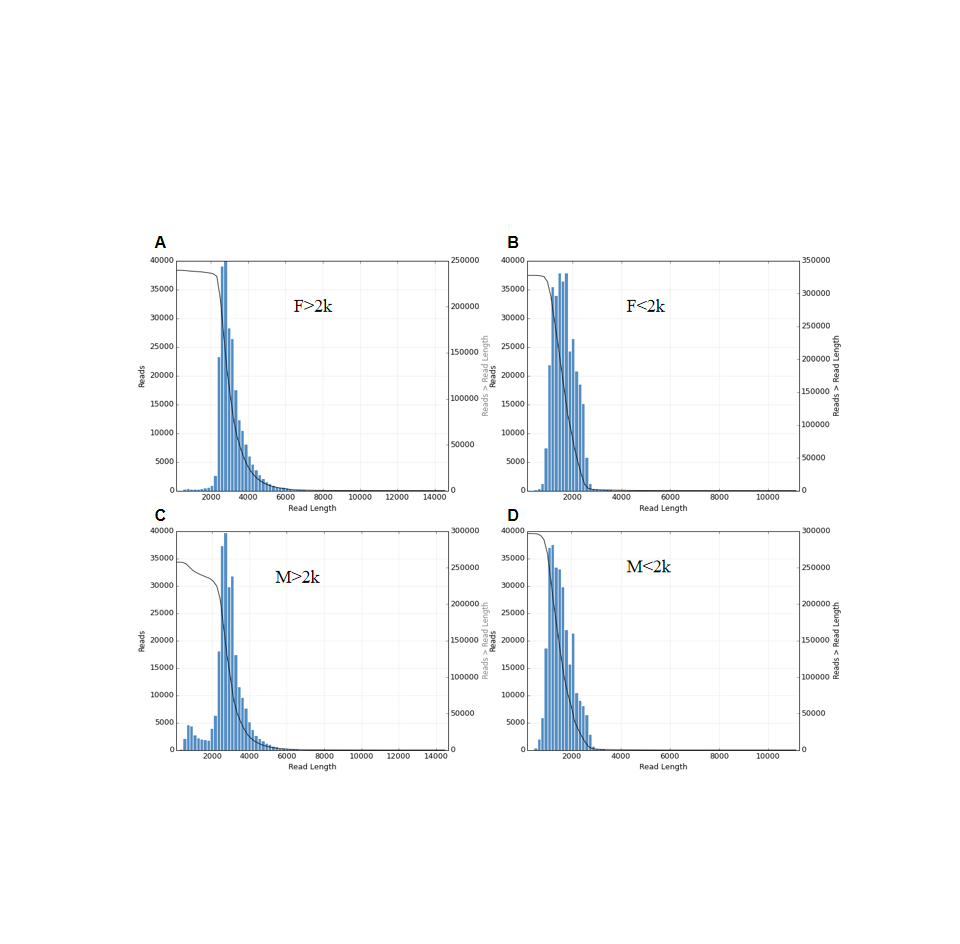

Supplement: Supplementary file 1 [file animals-13-03704-s001.zip › Figure S2.png]

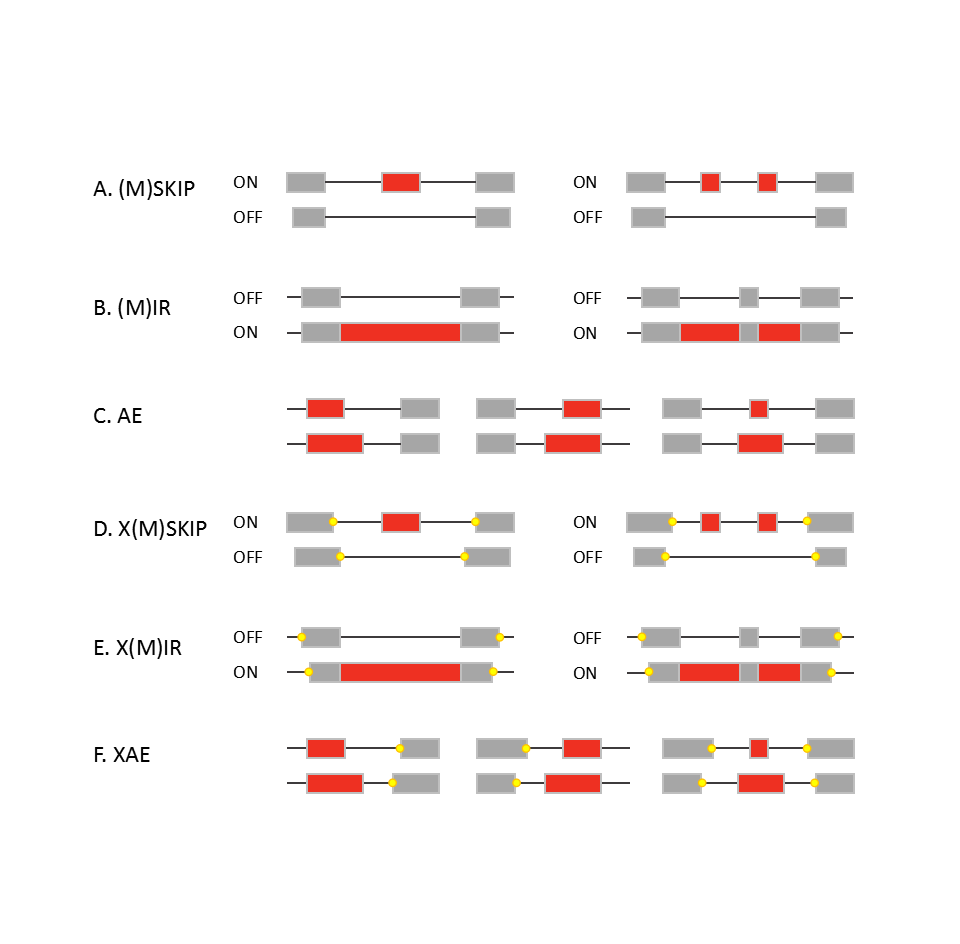

Supplement: Supplementary file 1 [file animals-13-03704-s001.zip › Figure S3.png]

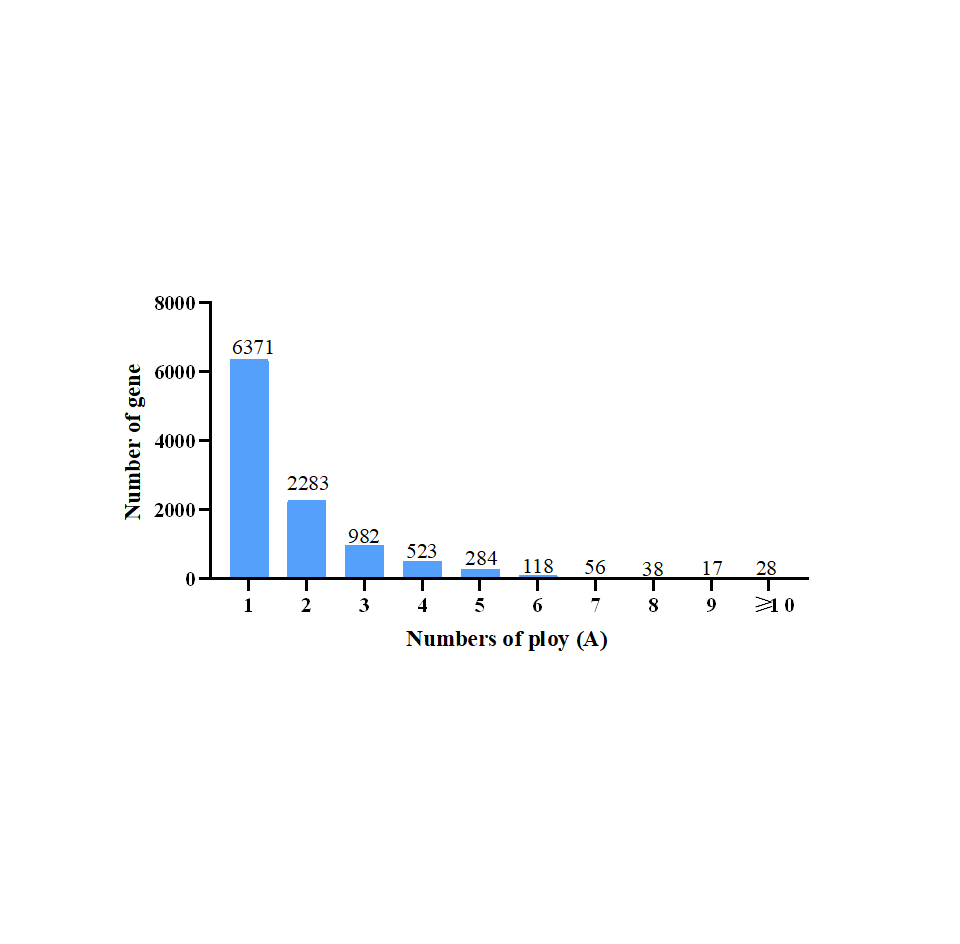

Supplement: Supplementary file 1 [file animals-13-03704-s001.zip › Figure S4.png]
